# Integrating 3-omics data analyze rat lung tissue of COPD states and medical intervention by delineation of molecular and pathway alterations

**DOI:** 10.1042/BSR20170042

**Published:** 2017-06-21

**Authors:** Jiansheng Li, Peng Zhao, Liping Yang, Ya Li, Yange Tian, Suyun Li, Yunping Bai

**Affiliations:** 1 Henan Key Laboratory of Chinese Medicine for Respiratory Disease, Henan University of Chinese Medicine, Zhengzhou, Henan 450046, China; 2 Collaborative Innovation Center for Respiratory Disease Diagnosis and Treatment and Chinese Medicine Development of Henan Province, Henan University of Chinese Medicine, Zhengzhou, Henan 450046, China; 3 Department of Respiratory Diseases, The First Affiliated Hospital of Henan University of Chinese Medicine, Zhengzhou 450000, China

**Keywords:** chronic obstructive pulmonary disease, metabolomics, molecular pathogenesis, proteomics, transcriptomics

## Abstract

Chronic obstructive pulmonary disease (COPD) is a serious health problem. However, the molecular pathogenesis of COPD remains unknown. Here, we explored the molecular effects of cigarette smoke and bacterial infection in lung tissues of COPD rats. We also investigated therapeutic effects of aminophylline (APL) on the COPD rats and integrated transcriptome, proteome, and metabolome data for a global view of molecular mechanisms of COPD progression. Using molecular function and pathway analyses, the genes and proteins regulated in COPD and APL-treated rats were mainly attributed to oxidoreductase, antioxidant activity, energy and fatty acid metabolism. Furthermore, we identified hub proteins such as Gapdh (glyceraldehyde-3-phosphate dehydrogenase), Pkm (pyruvate kinase isozymes M1/M2), and Sod1 (superoxide dismutase 1), included in energy metabolism and oxidative stress. Then, we identified the significantly regulated metabolic pathways in lung tissues of COPD- and APL-treated rats, such as arachidonic acid, linoleic acid, and α-linolenic acid metabolism, which belong to the lipid metabolism. In particular, we picked the arachidonic acid metabolism for a more detailed pathway analysis of transcripts, proteins, and metabolites. We could observe an increase in metabolites and genes involved in arachidonic acid metabolism in COPD rats and the decrease in these in APL-treated rats, suggesting that inflammatory responses were up-regulated in COPD rats and down-regulated in APL-treated rats. In conclusion, these system-wide results suggested that COPD progression and its treatment might be associated with oxidative stress, lipid and energy metabolism disturbance. Additionally, we demonstrated the power of integrated omics for the elucidation of genes, proteins, and metabolites’ changes and disorders that were associated with COPD.

## Introduction

Chronic obstructive pulmonary disease (COPD) is pathophysiologically characterized by chronic airflow limitation and progressive lung function decline resulting from an abnormal inflammatory response to inhaled particles and gases in the lung [1]. Although extensive investigations of COPD have taken place over the last few decades, its pathogenesis is still unclear [2]. Currently no treatment is available to prevent or halt the progression of these disorders [3,4]. Thus, we still need to gain deeper insights into their molecular pathogenesis and develop novel therapeutic strategies. However, pathologic changes result from intricate molecular network alterations, such as molecular links between subcellular components and disease genes, rather than form a few key genes or other functionally important biomolecules. Naturally, based on network thinking, identification of deregulated networks and pathways is an extremely effective discovery approach [5,6].

Transcriptomics-proteomics-metabolomics profiling techniques have proven to be powerful new tools for uncovering complex biological processes, which aid in exploration of novel mechanisms of disease pathogenesis and project future approaches to personalized medicine [7–9]. Transcriptomics studies have proven to be effective for exploring the entire genome. And the transcriptional networks govern temporally and spatially regulated gene expression programs. However, gene expression alone cannot provide the details of alternative splicing and protein expression. Proteomics makes it possible to provide the real information on the protein expression and protein function in a cellular context. Moreover, metabolomics provide a global view of the metabolic environment that is the consequence of the transcriptome and proteome [10,11]. Thus, integrative and comparative analyses of transcriptomics, proteomics and metabolomics datasets has a potential to give a detailed view of complex biological processes, such as the deregulated networks and pathways of COPD [9,12,13].

Previously, we developed a stable experimental COPD model on rats, using a combination of cigarette smoke inhalation and repeated *Klebsiella pneumoniae* infections. The pathologic changes in COPD rat airway that are similar to those occurring in human COPD patients. Thus, COPD rat can serve as a useful animal model for human COPD pathologies, and may also be useful for serial sampling for COPD biomarker studies and studies of therapeutic targets [14]. In addition, aminophylline (APL), a complex of theophylline and ethylenediamine, is commonly used in treatment of exacerbations of COPD [15]. Its main pharmacological action is relaxation of bronchial smooth muscle. Moreover, APL can decrease IL-8, IL-17, and TNF-α levels in bronchoalveolar lavage fluid and inhibit expression of MUC5AC and TLR4 in airway and lung tissues in COPD rats [16]. Previously, we treated COPD rats with APL, and found that APL could significantly decrease expression levels of inflammatory mediators [17].

In the present study, we analyzed lung tissues of COPD rats on a global scale by integrating transcriptomics, proteomics, and metabolomics data streams. We showed, under these experimental settings, that this integrated analyses provided a global picture of molecular mechanisms of COPD pathogenesis, including deregulated networks and pathways. We further analyzed the effects of the COPD drug APL, commonly used in the treatment of COPD. Taken together, this integrating study could provide the information for mammalian tissues disease states of COPD and medical intervention in a preclinical setting.

## Materials and methods

### Chemicals and animals

APL was obtained from Shandong Xinhua Pharmaceutical Co., Ltd. (Shandong, China). *K. pneumoniae* (strain ID: 46114) was purchased from the National Center for Medical Culture Collection (CMCC, Beijing, China). Mayer’s hematoxylin and 1% eosin alcohol solution were purchased from MUTO PURE CHEMICALS (Tokyo, Japan). Thirty-two Sprague–Dawley rats (16 male and 16 female; 200 ± 20 g) were obtained from the Experimental Animal Center of Henan province (Zhengzhou, China). The animals were housed in a temperature (25 ± 2°C) and humidity (50 ± 10%)-controlled environment with a 12:12-h light/dark cycle. Feed and water were available *ad libitum*. All animals were handled with humane care throughout the experiment.

### COPD model and drug administration

The rats were placed in a closed box and exposed to the tobacco (Hongqi Canal® Filter tip cigarette; tobacco type, tar: 10 mg; nicotine content: 1.0 mg; carbon monoxide: 12 mg, Henan Tobacco Industry, Zhengzhou, China) smoke of eight cigarettes for 30 min, twice per day with 3 h smoke-free intervals during the first two weeks, and to the smoke of 15 cigarettes for 30 min, thrice per day with 3 h smoke-free interval from weeks 3 to 12. The *K. pneumoniae* dilution (6 × 10^8^ CFU/ml, 0.1 ml) was dropped in an alternate fashion into the rat’s nostrils every 5 days from weeks 1 to 8. At the end of week 8, two COPD rats were killed to collect the lung tissues to validate that this rat model was successful [14].

**Figure 1:**
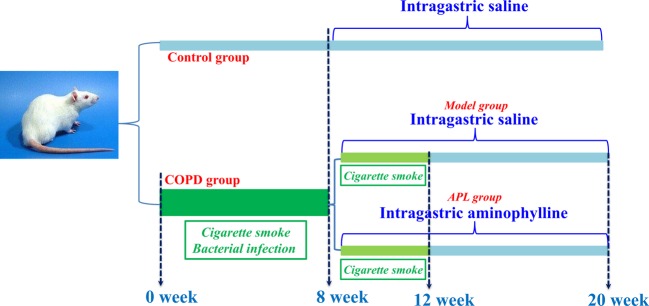
Establishment of a COPD rat model and drug administration. The flow chart explaining the COPD induction and drug administration regime.

In week 9, COPD rats were divided into two groups with ten rats each. Then, COPD rats were intragastrically treated with normal saline (2 ml) and APL (2.3 mg/kg) every day for 12 weeks. The control rats also were administrated intragastrically with normal saline (2 ml) for the same amount of time. All rats were killed at week 20 (Figure 1). The lung tissues were shock-frozen in liquid nitrogen and stored at –80°C before use. The experimental protocol was approved by the Experimental Animal Care and Ethics Committee of the First Affiliated Hospital, Henan University of Chinese Medicine. The animal experiments were conducted with approval of the Committee on the Care and Use of Laboratory Animals of the First Affiliated Hospital, Henan University of Chinese Medicine, China.

### Pulmonary function analysis

Pulmonary function data were detected. The changes of rat respiratory function were converted into electrical signals through a pressure transducer and amplifier and processed by a computer.

### Respiratory data collection

Respiratory function was evaluated by a sealed unrestrained whole body plethysmography (UWBP, Buxco Electronics, Troy, NY, U.S.A.) every fourth week from week 0 to 20. Rats were placed into the closed plethysmograph connected to a transducer and a computer. Finally, this work focussed on three measures: tidal volume (TV), peak expiratory flow (PEF) and 50% TV expiratory flow (EF50).

### Histological analyses

The lung tissues were fixed in neutral 10% buffered formaldehyde for 24 h, embedded in paraffin, sliced into 4-μm thick slices, and supplied for histological examination. Sections were stained with Mayer’s hematoxylin and then with 1% eosin alcohol solution (H&E staining). Samples were examined by Olympus BX51 microscope (Tokyo, Japan). Alveolar number, alveolar diameter, small pulmonary vessels bronchial wall thickness, and bronchiole stenosis scores were detected using Image Pro Plus® (IPP) 6.0 software (Media Cybernetics, MD, U.S.A.). Bronchia and lung injury scores were evaluated under an optical microscope.

### Gene expression analyses with microarrays

For transcriptomic analysis, lung tissue RNA was purified from six rats from each of the three experimental groups using a Qiagen RNeasy Micro Kit (Qiagen, Venlo, Netherlands). RNA integrity and quantity were verified using the Bioanalyzer 2100 (Agilent, Palo Alto, CA).

Total RNA was PCR amplified using First Strand cDNA Synthesis Kit (Roche, Basel, Switzerland) to conduct real-time PCR experiments, and labeled using Quick Amp kit (Agilent Technologies, Santa Clara, CA, U.S.A.) and hybridized with Agilent Whole Rat Genome Oligo Microarray (4 × 44 K) in Agilent’s SureHyb Hybridization Chambers. After hybridization, processed slides were scanned by an Agilent DNA microarray scanner (part number: G2505B) using manufacturer recommended settings. Samples from randomly chosen rats were analyzed using at least biological triplicates. All downstream microarray analyses were performed using Agilent GeneSpring GX software version 11.0. Microarray datasets were background subtracted and normalized by applying GeneSpring GX using the Agilent FE one-color scenario (mainly median normalization) Processed data were subsequently filtered through fold change (|log_2_ ratio|>1) and Student’s *t* test screening (*P*-value <0.05).

### Protein expression analysis

Lung tissue protein was purified from six rats from each of the three experimental groups. In brief, lysis solution (4% SDS, 0.1 M DTT, 0.1 M Tris pH 8.0) was added to each tissue and homogenized in a mechanical homogenizer (Retsch Technology, Haan, Germany). The homogenates were then subjected to needle sonication (Bandelin 2200 Ultrasonic homogenizer, Bandelin, Germany), and the total protein in the supernatant quantitated using a modified Bradford assay (Bio–Rad, Hercules, CA) according to manufacturer’s instructions. For trypsin digestion, trypsin (Roche, Mannheim, Germany) solution (protein/trypsin ratio 1:30) were added and incubated for 24 h. Labeling with iTRAQ eight-plex (AB SCIEX, Darmstadt, Germany) was performed for 2 h according to the manufacturer’s instructions. Each sample was dissolved in 0.1% FA and used for LC-MS analysis.

LC-MS/MS experiments were performed by Nano liquid chromatography (Daojing, Riben) coupled on-line to a Q-TOF mass spectrometer (Buluke). Peptide separation was performed on a pulled tip column (15 cm × 100 μm id) containing C18 Reprosil, 5 μm particles (Nikkyo Technos, Tokyo, Japan) using increasing amounts of acetonitrile containing 0.1% formic acid (mobile phase B) at 300 nl/min. Gradient conditions were: 5–34% B (0–25min), 34–60% B (25–30 min), 80% B held for 4 min, 80–5% B (1 min).

We used the loess and global median normalization to process the proteomics data. Data were log_2_ transformed and analyzed on both peptide and protein level. Statistical significance of observed fold-change ratios was determined by one sample *t* test. To select differentially expressed proteins for further validation, two criteria were applied in parallel: (i) fold change higher than 1.0 for up-regulation or lower than 1.0 for down-regulation, (ii) *P*-values <0.05 were considered as statistically significant for proteins.

### Metabolic profiling analysis

The lung tissue (100 mg) mixed with 1 ml of cold methanol/water (4:1, v:v) was homogenized using a high speed blender. After ultrasonication, the mixture was placed on ice for 20 min and then centrifuged for 10 min at a rotation speed of 20000×***g***. After that, 800 μl of supernatant was transferred and lyophilized in a freeze dryer and redissolved in 100 ml of methanol/water (4:1, v:v) before analysis.

Metabolic profiling of lung tissues was conducted on an Agilent-1200 LC system coupled to an Agilent-6520 Q-TOF mass spectrometry. Chromatographic separation was performed on an Eclipse plus C18 column (1.8 μm, 3.0 × 100 mm^2^, Agilent) with temperature of the column set at 40°C. The flow rate was 0.3 ml/min, the mobile phase was ultrapure water with 0.1% formic acid (A), and acetonitrile with 0.1% formic acid (B). The chromatographic elution procedure was performed at 40°C as follows: 0 min, 1% B; 1 min, 1% B; 3 min, 45% B; 9 min, 80% B; 11 min, 100% B; 18 min, 100% B; 19 min, 1% B; 25 min, 1% B. The sample injection volume was 5 μl. The parameters of mass detection were set as followed: drying gas (N_2_) flow rate, 10 l/min; gas temperature, 330°C; the nebulizer gas pressure, 40 psig; capillary voltage was 4000 V in positive mode and 3000 V in negative mode; fragmentor, 135 V; skimmer, 65 V; scan range was from m/z 100 to 1000 [18].

The LC-MS raw MS data were exported by Agilent Mass Hunter Qualitative Analysis Software (Agilent Technologies, Palo Alto, CA, U.S.A.). Before multivariate analysis, the data of each sample were normalized to total area to correct for MS response shift between injections due to any possible intra- and interday variations. The total integrated area of each sample was normalized to 1000. Partial least squares discriminant analysis (PLS-DA) in software SIMCA-P (ver 11.0, Umetrics, Umea, Sweden) was used for metabolite profile analysis [19]. Significance was determined using the Student’s paried *t*test and the one-way ANOVA on the mean of three different experiments. *P*-values less than 0.05 were considered significant.

### Gene/protein set enrichment, network, and pathway analyses

RNA expression data and protein were analyzed using KEGG database from Molecular Signature Database (MSigDB). We considered regulated pathways only as statistically significant, if the FDR was ≤1. For correlation analyses on pathway levels, we compared the KEGG pathway regulation, and we included only regulated pathways with FDR ≤1 for RNA or protein. Correlation analyses were done separately. We applied BINGO, a Cytoscape plugin, to explore the molecular function of genes and proteins [20].

To identify the main hub proteins, we generated interaction networks using STRING 10. We applied a high confidence score of 0.7, which indicated that only interactions with a high level of confidence were extracted from the database and considered as valid links for protein–protein interaction (PPI) networks.

Interaction networks were further analyzed using the Cytoscape v3.1.1 plugin called ‘network analyzer’ [21,22]. We used network analyzer to evaluate the hub proteins by analyzing the highest closeness centrality (CC), betweenness centrality (BC), and the node degree.

We applied MetScape to analyze the integrated pathway of gene/protein expression and metabolomics data [23].

### Statistical analysis

Differences between groups were determined by one-way ANOVA with the SPSS 19.0 software package (SPSS, Chicago, IL, U.S.A.). Values are expressed as means ± S.E.M. For all the tests, a two-sided *P*-value less than 0.05 was considered significant.

## Results and discussion

### Pulmonary function and pathology of COPD

**Figure 2:**
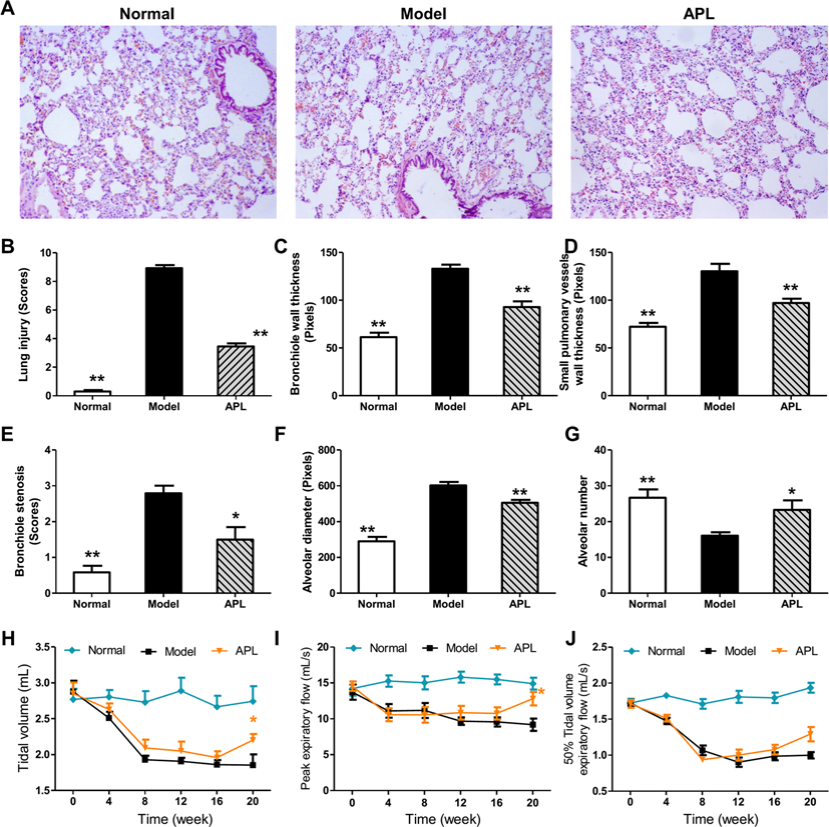
Histological changes in lung tissues of COPD rats and the therapeutic efficacies of APL A rat model of COPD was induced by cigarette smoke and bacterial infection. The COPD rats were intragastrically treated with APL (2.3 mg/kg) once daily. Control and model rats were treated with normal saline. Histopathologic changes of the lung tissues were detected on week 20 (H&E staining, magnification: ×100) (**A**). The lung injury scores were analyzed (**B**). Small pulmonary vessels wall thickness (**C**), bronchial wall thickness (**D**), bronchiole stenosis (**E**), alveolar diameter (**F**), and alveolar number (**G**) were detected. TV (**H**), PEF (**I**), and EF50 (**J**) were analyzed every fourth week from week 0 to 20. Results were given as means ± S.E.M., *n*=10. *P<0.05, **P<0.01 compared with model.

In order to clarify molecular details of the perturbations in lung tissues of COPD, we established a rat model of cigarette smoke- and bacterial infection induced COPD. We further treated COPD rats with bronchodilator APL to improve the airflow limitation. The pathological parameters including lung histopathology and mechanics confirmed cigarette smoke- and bacterial infection induced COPD, as well as the effects of APL treatment were shown in Figure 2. Compared with the control group, lung injury scores, bronchiole wall thickness, small pulmonary vessels wall thickness, bronchiole stenosis, and alveolar diameter were increased in model rat. The increase in lung injury scores, bronchiole wall thickness, small pulmonary vessels wall thickness, bronchiole stenosis, and alveolar diameter in COPD rats were clearly suppressed by APL treatment, compared with the COPD rats (Figure 2A–F). The main pharmacological action of APL is relaxation of bronchial smooth muscle [16]. Here, APL treatment also increased the alveolar number in COPD rats (Figure 2G). In addition, compared with the model rats, TV, PEF, and EF50 clearly decreased in the model rat from weeks 4 to 20, whereas, APL significantly increased the TV and PEF, and slightly increased the EF50 in COPD rats at week 20 (Figure 2H–J). Moreover, some of these COPD rats were intragastrically administrated with normal saline and APL (2.3 mg/kg) every day from week 9 to 20, and then killed in week 32. Pulmonary function were analyzed every fourth week from weeks 0 to 32. In this result, we also found that APL significantly increased the TV and PEF and slightly increased the EF50 in COPD rats during week 20 to 32. These data indicated that APL could improve pulmonary function after 12 weeks of treatment (week 20) (Supplementary Figure S1). The results demonstrated that pathologic changes in COPD rat airways were similar to those occurring in COPD patients [2,24], and APL treatment could effectively prevent COPD.

### Alterations of molecules on the transcriptome level

**Figure 3:**
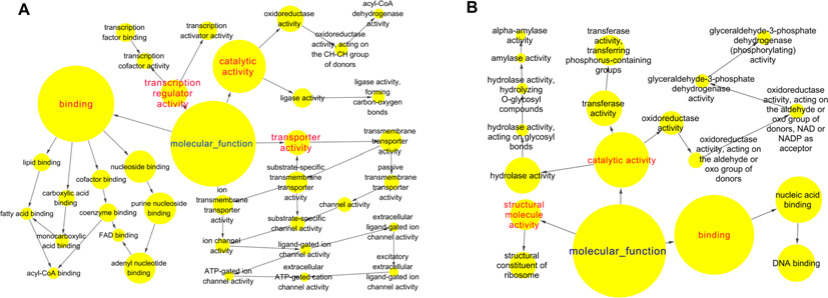
Molecular functions of regulated genes in lung tissues of COPD and APL-treated rats Functionally grouped network of enriched categories was generated for the regulated genes using BINGO. The area of a node is proportional to the number of genes in the test set annotated to the corresponding GO category. (**A**) Representative molecular function of regulated genes in lung tissues of COPD rats. (**B**) Representative molecular function of regulated genes in lung tissues of APL-treated rats.

To identify RNA expression in COPD, we performed a microarray-based RNA expression study on the lung tissues, and approxiamtely 41000 genes were identified. In these datasets, we then identified 2463 and 2130 genes whose *P*-value was <0.05 regulated in COPD model compared with control and APL treatment compared with COPD model, respectively. These transcripts regulated in COPD model (compared with control) can be attributed to various molecular functions such as regulation of oxidoreductase activity, channel activity, fatty acid binding, glucose or fatty acid metabolism (Figure 3A), which are the activities mainly responsible for COPD. Similarly, the large majority of transcripts regulated in APL-treated rats were related to oxidoreductase activity and nucleic acid binding (Figure 3B).

In COPD model group, the regulated genes could be mapped to many pathways, such as fatty acid metabolism, p53 signaling pathway and cell adhesion molecules (Table 1). The genes regulated in APL treatment group could be mapped to adherens junction, MAPK signaling pathway, glycerophospholipid metabolism, Wnt signaling pathway, etc. (Table 2).

**Table 1:** Analyzed pathways of transcriptomics data regulated in lung tissue of COPD rats

Pathway	Total	*P*-value	FDR
Valine, leucine, and isoleucine degradation	15	1.43E-06	0.0017
Propanoate metabolism	12	7.33E-06	0.0089
Ribosome	19	1.02E-05	0.0124
Endocytosis	30	1.06E-04	0.1291
Fatty acid metabolism	11	4.45E-04	0.5402
Graft compared with host disease	12	0.00058	0.7029
Viral myocarditis	16	0.000803	0.9725
Antigen processing and presentation	16	0.000908	1.0993
Type I diabetes mellitus	12	0.002375	2.8508
Allograft rejection	11	0.002977	3.5612

Abbreviation: FDR, false dicovery rate.

**Table 2:** Analyzed pathways of transcriptomics data regulated in lung tissue of APL-treated rats

Term	Total	*P*-value	FDR
GnRH signaling pathway	13	0.007279	8.4431
Ribosome	12	0.007577	8.7746
Alzheimer’s disease	21	0.00908	10.4295
Adherens junction	11	0.00934	10.7131
Endocytosis	21	0.011227	12.7442
MAPK signaling pathway	24	0.032266	32.7012
Huntington’s disease	18	0.045681	43.1407

Abbreviation: FDR, false dicovery rate.

More information oo the regulated transcripts is shown in Supplementary Tables S1 and S2. The highlighted gene transcripts might serve as a starting point for further biological pathway information analysis and potentially represent candidates for biomarker exploration.

### Alterations of molecules on the proteome level

**Figure 4:**
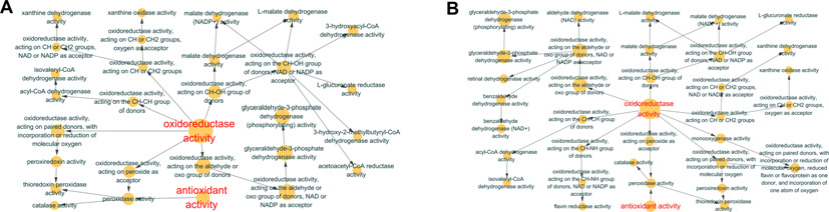
Molecular functions of regulated proteins in lung tissues of COPD and APL-treated rats Functionally grouped network of enriched categories was generated for the regulated proteins using BINGO. The area of a node is proportional to the number of genes in the test set annotated to the corresponding GO category. (**A**) Representative molecular function of regulated proteins in lung tissues of COPD rats. (**B**) Representative molecular function of regulated proteins in lung tissues of APL-treated rats.

We next sought to examine their expression profiles at the protein level, and identified 191 and 187 proteins regulated in COPD model compared with control and APL treatment compared with COPD model, respectively (Supplementary Tables S3 and S4). The COPD model group (191 proteins) shared 94 common proteins with the APL treatment group (187 proteins). Out of the 94 proteins, expression changes of 54 proteins in COPD model was down-regulated by APL treatment, suggesting these proteins might be related to the therapeutic effect of APL. The proteins regulated in COPD model and APL-treatment group could be attributed to similar molecular functions, such as oxidoreductase activity and antioxidant activity (peroxidase activity, catalase activity, and thioredoxin peroxidase activity) (Figure 4A,B).

**Figure 5:**
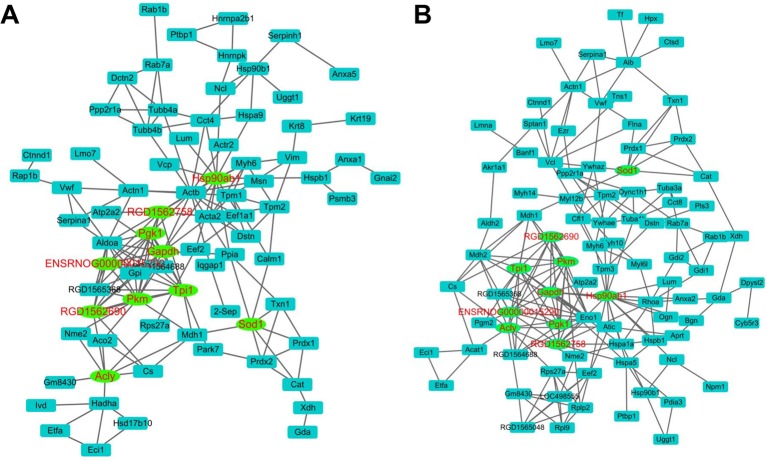
The networks of proteins constructed by STRING and Cytoscape. Network of regulated proteins in lung tissues of COPD (**A**) and APL-treated rats (**B**), analyzed by STRING (version 10) and Cytoscape software. The protein interaction networks were generated by STRING 10, then analyzed and visualized by Cytoscape. Nodes were connected proteins within the network. The top hub proteins were indicated (green) after removal of a node.

The KEGG pathway affiliation of these proteins revealed that the proteins regulated in COPD and APL-treated rats belong to energy metabolism (e.g. glycolysis/gluconeogenesis and pyruvate metabolism), fatty acid metabolism, tight junction etc. (Tables 3 and 4). Furthermore, using the STRING 10 and Cytoscape, we analyzed protein networks to identify the hub proteins that may exert important functions in COPD and APL-treated rats. As shown in Figure 5, we used the Cytoscape plug in ‘network analyzer’ and a robustness test to identify the hubs. The main important proteins in both networks were Hsp90ab1 (heat shock protein HSP 90-β), Pkm (pyruvate kinase isozymes M1/M2), Tpi1 (Tpi1 protein), Gapdh (glyceraldehyde-3-phosphate dehydrogenase), RGD1562758 (Gapdh), ENSRNOG00000015290 (triosephosphate isomerase), Acly (ATP-citrate synthase isoform 1), Pgk1 (phosphoglycerate kinase 1), RGD1562690 (L-lactate dehydrogenase A chain), Sod1 (superoxide dismutase 1) (Supplementary Tables S5 and S6). The results revealed that the important proteins in the networks belonged to energy metabolism and oxidative stress.

**Table 3:** Analyzed pathways of proteomics data regulated in lung tissue of COPD rats

Term	Total	*P*-value	FDR
Glycolysis/gluconeogenesis	10	6.64E-06	0.0074
Hypertrophic cardiomyopathy (HCM)	9	5.29E-05	0.0589
Dilated cardiomyopathy	9	8.70E-05	0.0969
Pyruvate metabolism	6	3.62E-04	0.4022
Glyoxylate and dicarboxylate metabolism	4	7.99E-04	0.8869
Tight junction	9	0.001079	1.1955
Citrate cycle (TCA cycle)	5	0.00125	1.3842
Leukocyte transendothelial migration	8	0.002374	2.6132
Focal adhesion	10	0.003903	4.2629
Tryptophan metabolism	5	0.004803	5.222
Valine, leucine, and isoleucine degradation	5	0.006122	6.6129
Adherens junction	6	0.00651	7.0181
Cardiac muscle contraction	6	0.008115	8.6774
Propanoate metabolism	4	0.015539	16.0102
Prion diseases	4	0.018217	18.5205
Fatty acid metabolism	4	0.029489	28.3564
Arrhythmogenic right ventricular cardiomyopathy (ARVC)	5	0.030762	29.3969
Purine metabolism	7	0.03707	34.3504

Abbreviation: FDR, false discovery rate.

**Table 4:** Analyzed pathways of proteomics data regulated in lung tissue of APL-treated rats

Term	Total	*P*-value	FDR
Tight junction	10	1.82E-04	0.1969
Focal adhesion	12	2.14E-04	0.231
Glycolysis/gluconeogenesis	8	3.20E-04	0.3449
Pyruvate metabolism	6	3.26E-04	0.3514
Regulation of actin cytoskeleton	11	0.001517	1.627
Leukocyte transendothelial migration	8	0.002086	2.2319
ECM–receptor interaction	6	0.008647	8.9587
HCM	6	0.010044	10.3356
Citrate cycle (TCA cycle)	4	0.011261	11.5206
Metabolism of xenobiotics by cytochrome P450	5	0.01431	14.4252
Glyoxylate and dicarboxylate metabolism	3	0.014596	14.6931
Prion diseases	4	0.017161	17.0621
Drug metabolism	5	0.026207	24.9496
Adherens junction	5	0.028629	26.9431
Tryptophan metabolism	4	0.029584	27.7156
Purine metabolism	7	0.033727	30.9815
Glutathione metabolism	4	0.043462	38.1359

Abbreviation: FDR, false discovery rate.

### Alterations of molecules on the metabolome level

**Figure 6:**
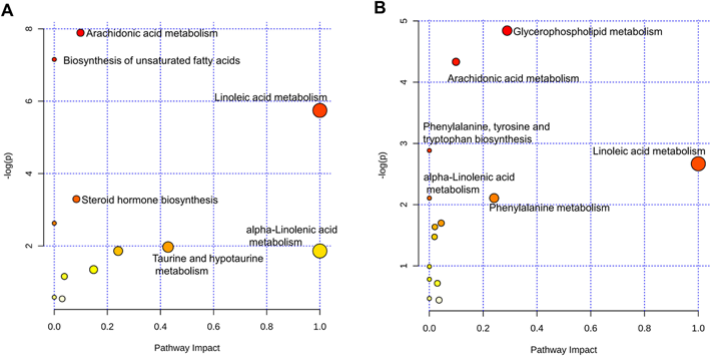
Metabolomic pathway analysis with MetaboAnalyst. Pathway analysis of the metabolites in lung tissues of COPD (**A**) and APL-treated rats (**B**). On the basis of all differential metabolites (Supplementary Tables S7 and S8 in the Supplementary material), global metabolic disorders of the most relevant pathways were revealed using the MetaboAnalyst. A Google-map style interactive visualization system was implemented to facilitate data exploration and generate pathway views.

Finally, to characterize the metabolic profile of COPD rats, the levels of metabolites were detected by LC-MS. Compared with those in healthy control rats, 49 metabolites were identified in COPD model rats. After treatment with APL, compared with those in COPD model rats, 32 metabolites were identified in APL-treated rats (Supplementary Tables S7 and S8). To have a better understanding of these metabolites, MetaboAnalyst was used to study the overview of systematic metabolome changes based on pathway analysis. The metabolic perturbation was analyzed from the perspective of pathway enrichment analysis combined with topology analysis. The most relevant pathways on the basis of metabolites were shown in Figure 6A,B. The perturbation of arachidonic acid and linoleic acid metabolism was considered to be responsible for COPD. More importantly, insights into the underlying pathogenesis of COPD could be provided from pathways regarding defined biomarkers (Tables 5 and 6). These metabolic anomalies were primarily involved in lipid metabolism, which are discussed in detail later.

**Table 5:** Analyzed pathways of metabolomics data regulated in lung tissue of COPD rats

	Total	Expected	Hits	Raw *P*	log *P*	FDR
Arachidonic acid metabolism	36	0.6676	5	0.0004	7.8918	0.030278
Biosynthesis of unsaturated fatty acids	42	0.7789	5	0.0008	7.1542	0.031654
Linoleic acid metabolism	5	0.0927	2	0.0032	5.7455	0.086323
Steroid hormone biosynthesis	70	1.2981	4	0.0371	3.2955	0.75027
Phenylalanine, tyrosine, and tryptophan biosynthesis	4	0.0742	1	0.0722	2.6281	1
Taurine and hypotaurine metabolism	8	0.1484	1	0.1394	1.9704	1
Phenylalanine metabolism	9	0.1669	1	0.1555	1.8614	1
α-Linolenic acid metabolism	9	0.1669	1	0.1555	1.8614	1
Glyoxylate and dicarboxylate metabolism	16	0.2967	1	0.2600	1.3470	1
Citrate cycle (TCA cycle)	20	0.3709	1	0.3141	1.1582	1
Fatty acid biosynthesis	43	0.7974	1	0.5584	0.5827	1
Primary bile acid biosynthesis	46	0.8531	1	0.5833	0.5391	1

**Table 6:** Analyzed pathways of metabolomics data regulated in lung tissue of APL-treated rats

	Total	Expected	Hits	Raw *P*	log *P*	FDR
Glycerophospholipid metabolism	30	0.5136	3	0.0132	4.3261	0.88009
Arachidonic acid metabolism	36	0.6163	3	0.0217	3.8290	0.88009
Phenylalanine, tyrosine, and tryptophan biosynthesis	4	0.0685	1	0.0668	2.7060	1
Linoleic acid metabolism	5	0.0856	1	0.0828	2.4910	1
α-Linolenic acid metabolism	9	0.1541	1	0.1443	1.9358	1
Phenylalanine metabolism	9	0.1541	1	0.1443	1.9358	1
Glycosylphosphatidylinositol (GPI)-anchor biosynthesis	14	0.2397	1	0.2156	1.5342	1
Selenoamino acid metabolism	15	0.2568	1	0.2292	1.4732	1
Pantothenate and CoA biosynthesis	15	0.2568	1	0.2292	1.4732	1
Glycerolipid metabolism	18	0.3081	1	0.2685	1.3147	1
Aminoacyl-tRNA biosynthesis	67	1.1469	2	0.3197	1.1404	1
Steroid hormone biosynthesis	70	1.1983	2	0.3390	1.0817	1
Alanine, aspartate and glutamate metabolism	24	0.4108	1	0.3416	1.0743	1
Glycine, serine, and threonine metabolism	32	0.5478	1	0.4281	0.8484	1
Biosynthesis of unsaturated fatty acids	42	0.7190	1	0.5210	0.6520	1
Primary bile acid biosynthesis	46	0.7875	1	0.5540	0.5906	1
Purine metabolism	68	1.1641	1	0.6998	0.3569	1

### Relation between transcriptome, proteome and metabolome levels

As discussed above, we detected many genes, proteins, and metabolites, which possessed extensive biological function of complex physiological processes in COPD rats and during drug treatment. Next, we sought to analyze molecular network in lung tissues of COPD and medical intervention in a preclinical setting by integrating transcriptomics, proteomics, and metabolomics data.

However, making a direct correlation between mRNA, protein and metabolome levels is hampered by many difficulties [25–27]. The systems biological interpretation of the gene, protein, and metabolite measurements might be more adequately described on the system level rather than focussed excessively on individual molecules [6,28].

**Figure 7:**
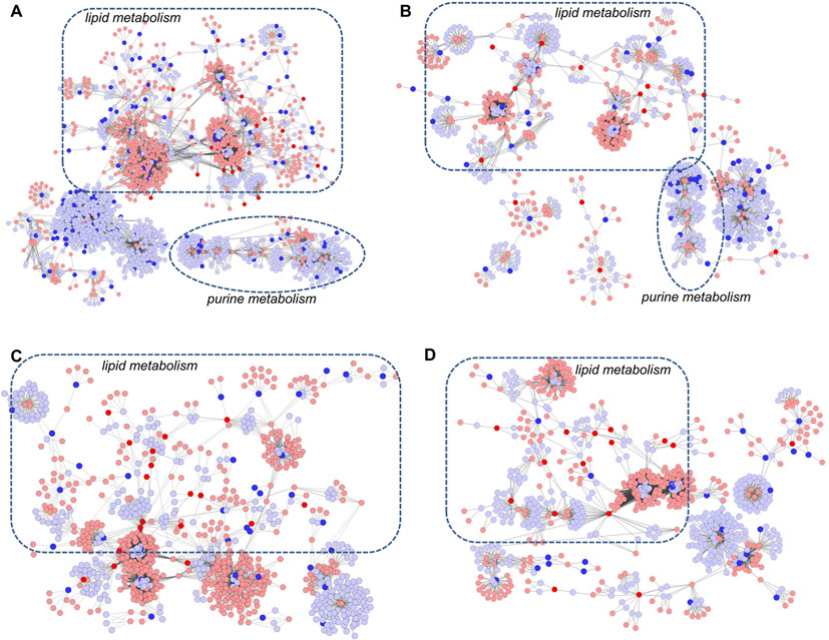
Metabolic correlation networks of the differential metabolites and genes (proteins) The compound reaction network with compounds (hexagons) and metabolic enzymes (rounds) as nodes and reactions as edges was constructed by MetScape. Input compounds were shown in blue, while input genes and proteins were shown in red. (**A**) Network of metabolites and genes regulated in lung tissues of COPD rats. (**B**) Network of metabolites and genes regulated in lung tissues of APL-treated rats. (**C**) Network of metabolites and proteins regulated in lung tissues of COPD rats. (**D**) Network of metabolites and proteins regulated in lung tissues of APL-treated rats.

To investigate the latent relationships of metabolite and gene (protein) measurements, a correlation network diagram was constructed using the MetScape software (Figure 7) [23]. All the metabolite and gene (protein) measurements were involved in the diagram to obtain a global view of complex physiological processes in COPD rats and during drug treatment.

As shown in Figure 7A, the correlation networks were constructed based on the transcriptome and metabolome information of COPD rats. We observed that gene—metabolite networks could be divided into three primary groups: lipid metabolism, purine metabolism, and phosphoprotein. A number of genes and all the metabolite measurements of COPD rats were included in lipid metabolism. Similarly, many genes and most of metabolite measurements of drug treatment rats also were included in lipid metabolism (Figure 7B).

Then, we constructed the protein–metabolite networks based on the metabolite and protein measurements of COPD rats (Figure 7C) and drug-treated rats (Figure 7D). The results showed that both the networks consisted of two main components: lipid metabolism and purine metabolism. In these networks, more than half of proteins and almost all the metabolites could be mapped to lipid metabolism.

These results implied that lipid metabolism disorder play important roles in the pathogenesis of COPD, and APL could provide therapeutic effects on COPD by regulating lipid metabolism.

To investigate the latent relationships of the transcript and protein measurements, we further analyzed the overlapping pathways between transcript and protein measurements. These transcripts and proteins of COPD rats could be mapped into three common pathways, such as fatty acid metabolism, propanoate metabolism, and valine, leucine and isoleucine degradation, which could be attributed to lipid metabolism, energy metabolism and amino acid metabolism (Tables 1,3,7). However, we found two common genes (proteins) in these pathways. The two genes make two corresponding proteins: acetyl-CoA acetyltransferase (mitochondrial), trifunctional enzyme subunit α (mitochondrial). The results of integrated pathway analysis revealed that regulation of lipid metabolism, amino acid metabolism, and energy metabolism might be the important biological function of complex physiological processes in COPD rats.

**Table 7:** The common pathway amongst the genes, proteins, and metabolites

Term		Gene
Valine, leucine, and isoleucine degradation	Proteomics	HSD17B10, IVD, ALDH2, **ACAT1, HADHA**
	*Transcriptomics*	*BCKDHA, ALDH6A1, ACADM, HEATR7B2, RGD1562373, ECHS1, **ACAT1, HADHA**, AUH, RGD1564209, ALDH7A1, HMGCS2, OXCT1, ALDH1A7, HADH, PCCB, ACAA1*
Propanoate metabolism	Proteomics	LOC365605, LDHA, LOC502220, LOC366627, LOC502627, LOC366355, ALDH2, LOC503477, **ACAT1, HADHA**, LOC364462
	*Transcriptomics*	*ALDH6A1, ACADM, SUCLG2, ECHS1, **ACAT1***, **HADHA** *VSX1, ACSS1, ALDH7A1, MLYCD, ALDH1A7, SUCLA2, PCCB*
Fatty acid metabolism	Proteomics	ALDH2, **ACAT1**, DCI, **HADHA**
	*Transcriptomics*	*ACADVL, ACOX1, ALDH7A1, ACADM, RGD1562373, ECHS1, ALDH1A7, HADH, PECI, **ACAT1, HADHA**, ACAA1*
Arachidonic acid metabolism	*Transcriptomics*	*LTA4H, PTGES*
	*Metabolites*	*5-HPETE, LTA4, 5-HETE, 20-OH-LTB4, LXA4, PGE2*

Bold text represent two common genes (proteins_ in these pathways

However, the transcripts and proteins of drug-treated rats were only mapped into a common pathway: adherens junction (Tables 2 and 4). This weak pathway correlation could be explained in part by differential regulation and life times of RNAs and proteins.

### Arachidonic acid metabolism: a typical example of the transcript, protein, and metabolite datasets

**Figure 8:**
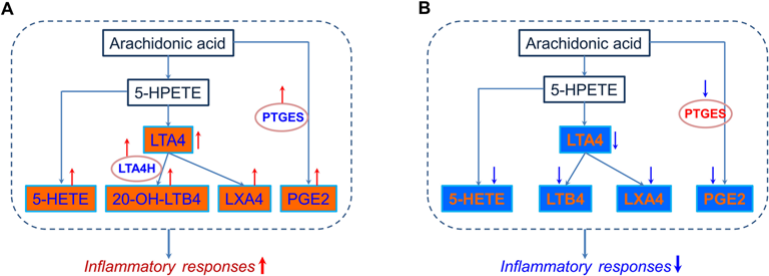
Pathway analysis of transcripts, proteins and metabolites in arachidonic acid metabolism. Simplified arachidonic acid metabolism featuring transcriptomics and metabolomics regulations in lung tissue of COPD (A) and APL-treated (**B**) rats, according to KEGG nomenclature. Transcriptomics data are presented as ovals and metabolites data as rectangles. Regulation is color coded in which red stands for up-regulated (red, ↑), blue for down-regulated (blue, ↓), and black for unregulated.

We strengthened pathway analyses of transcript, protein, and metabolite datasets, and found that a number of transcripts and proteins and almost all the metabolites could be mapped to lipid metabolism. In addition, in the pathway enrichment analysis of metabolites, we found that arachidonic acid metabolism was a strongly regulated pathway. Thus, for a more detailed pathway analysis of transcripts, proteins, and metabolites, we picked arachidonic acid metabolism as a typical regulated pathway (Figure 8). As shown in Figure 8 A, six metabolites and two transcripts of COPD rats were included in arachidonic acid metabolism, and these transcripts and metabolites were up-regulated, which suggested that arachidonic acid metabolism pathway was activated, and the expression levels of inflammatory mediators such as PGE2 were increased (Table 7). Five down-regulated metabolites and one down-regulated transcript of drug-treated rats were mapped into arachidonic acid metabolism, which suggested that arachidonic acid metabolism and the production of inflammatory mediators were inhibited by APL (Figure 8B). However, protein measurements were not included in arachidonic acid metabolism, and these proteins might participate in regulating the upstream or downstream components of arachidonic acid metabolism pathway.

## Conclusion

Integrating transcriptomics, proteomics, and metabolomics data is becoming more important to analyze in mammalian tissues disease states and medical intervention in a preclinical setting. However, attempts thus far to combine omics data streams have been met with limited success.

In our study, we detected a number of RNA-, protein-, and metabolite-based biomarker candidates. In particular, we observed regulation of oxidoreductase activity, channel activity, antioxidant activity, fatty acid binding, glucose or fatty acid metabolism in lung tissues of COPD rats and APL-treated rats. Consistently, the results of combined analyses of three datasets demonstrated that lipid metabolism were the critical pathways in COPD rats and APL-treated rats. Moreover, we found that the arachidonic acid metabolism was a strongly regulated pathway. In this pathway, we observed up-regulation of the metabolites (20-HETE, 5-HETE, LTA4, 20-OH-LTB4, LXA4 and PGE2) and metabolic enzymes (LTA4H (leukotriene A4 hydrolase), PTGS2 (prostaglandin-endoperoxide synthase 2)) in COPD rats and down-regulation of the metabolites (5-HETE, LTA4, LTB4, and LXA4) and metabolic enzymes (PTGS1/2/3) involved in APL-treated rats. A large number of researches indicated that LXA4 and LXB4 promote the resolution of inflammation. LXA4 and LXB4 could inhibit neutrophil chemotaxis, eosinophil trafficking and transmigration, generation of superoxide anions by neutrophils, and degranulation of azurophilic granules, and block natural killer cell cytotoxicity and tumor necrosis factor-α release from T cells [29–31]. In addition, many studies found LTB4, cysteinyl-containing LTs, and PGE2, PGF2 α, 6-oxo-PGF1 α, and thromboxane B2 were found in sputum samples of COPD patients [32]. Using network and pathway analyses, we could demonstrate in lipid metabolism pathways, such as arachidonic acid metabolism, the pathological changes of COPD rats and specific restoring effects of APL treatment in lung tissues. However, the major limitation of this approach is that identified genes, proteins and metabolites need to be further experimentally validated as marker sets for targetted and integrated transcriptomics, proteomics, and metabolomics approaches.

## Abbreviations

APL: aminophylline
CFU: colony-forming units
COPD: chronic obstructive pulmonary disease
ECM: extracellular matrixc
EF50: 50% tidal volume expiratory flow
FA: formic acid
FDR: false discovery rate
Gapdh: glyceraldehyde-3-phosphate dehydrogenase
GO.: gene ontology
H&E: hematoxylin-eosin
IL-8: interleukin 8
KEGG: kyoto encyclopedia of genes and genomes
MUC5AC: mucin 5AC
PEF: peak expiratory flow
TV: tidal volume
TNF-α: tumor necrosis factor
TLR4: toll-like receptor 4
Q-TOF: quadrupole time-of-flight
